# 

**Published:** 1914-05-23

**Authors:** 


### Gifts and Bequests.

Mrs. Mary Ann Wingrove, of Southampton, has left £300 each to the Royal West of England Deaf and Dumb Institution, the West of England Institution for the Instruction and Employment of the Blind, and five other charities, and after certain legacies the residue of her estate, amounting to some £17,000, she left in equal shares to Dr. Barnardo's Homes, the Devon and Exeter Hospital, Exeter, the Royal South Hants and Southampton Hospital, Southampton, the Cancer Hospital, Brompton, and four other charities.

Mr. Alfred Benson, of Scarborough, formerly outdoor goods superintendent at York of the North Eastern Railway, has left £2,200 to charities, including £600 each to the York County Hospital.

Mrs. Mary Elizabeth Booth, of Redstone Park, Redhill, Surrey, has left £400 each to the London Hospital, the University College or North London Hospital, the Children's Hospital, Paddington Green, and St. Mary's Hospital, Paddington.

### Medical Appointments.

Dr. W. E. Robinson, M.D., B.Ch. Oxon., has been appointed honorary anæsthetist to the *Dreadnought* Hospital of the Seamen's Hospital Society at Greenwich.

Dr. J. G. Bennet, M.B., Ch.B., has been appointed medical officer to the out-patients' department of the Manchester Children's Hospital, Gartside Street, Manchester. Dr. M. E. May has been appointed assistant medical officer, and Dr. J. H. Wright, M.B., B.A. (Dublin), has been appointed second assistant medical officer at the out-patients' department, Gartside Street, Manchester.

The appended new medical appointments have been made at the Gravesend Hospital: Following the retirement of Mr. Pinching and Dr. Firth after over thirty years' honorary work the following gentlemen have been appointed on the hospital staff:—Mr. C. J. Pinching, B.A., M.B., B.Ch. Oxon., M.R.C.S., L.R.C.P. Lond., and Mr. J. D. Hartley, F.R.C.S. Eng., L.R.C.P. Lond., as honorary surgeons; Mr. S. M. Lawrence, M.D., B.S. Lond., L.R.C.P. Lond., M.R.C.S. Eng., as honorary assistant surgeon; and Mr. C. Dismorr, M.R.C.S. Eng., L.R.C.P. Lond., as honorary junior assistant surgeon.

### The Book Market.

LIST OF NEW BOOKS RECEIVED FROM THE FOLLOWING PUBLISHERS:—

The Scientific Press, Ltd.: "Massage Case Book," 6d. net; "Midwives' Pocket-book," New Edition, Honnor Morten, 1s.; "Complete Handbook of Midwifery," New Edition, J. K. Watson, 6s. net; "Ionic Medication" (S.P. Pocket Guide Series), "Theory and Practice," 1s. net.

Constable and Co.: "Outlines of Chordate Development," Wm. E. Kellicott, 10s. 6d. net; "Text-book of General Embryology," Wm. E. Kellicott, 10s. 6d. net.

Funk and Wagnalls Co.: "Exercises for Women," Florence Bolton, A.B.

John Murray: "Researches into Induced Cell-reproduction in Amœbæ," Cropper and Drew, 5s. net.

T. C. and E. C. Jack: "Cookery for Every Household," by Florence B. Jack, 3s. 6d. net.

Oxford Medical Press: "The Early Diagnosis of Tubercle." Riviere.

### Pamphlets and Papers.

"Printers' Pie," published by the *Sphere* and *Tatler*, Ltd. 1914. Price 1s.

"Spectacles for the Needy, and How to Get Them," by Sibyl Bristowe.

Treatment of Animals (the A.F. Pamphlet Series), being a Speech delivered at the Kensington Town Hall on December 15, 1913, at a meeting called to protest against cruelties to performing animals, by John Galsworthy. Animals' Friend Society, York House, Portugal Street, W.C. Price 2d.

### Institution and Public Reports.

Ninth Annual Report (for 1913) of the British Institute of Social Service.

The Lady Almoner's Report (March 1913-14) of St. Thomas's Hospital.

First Biennial Report of the Board of Control of State Institutions, ending June 30, 1912. [The Report is addressed to the Governor of North Dakota, U.S.A.]

Seventy-second Annual Report of the Royal Southern Hospital, Liverpool.

Twenty-ninth Annual Report (for 1913) of the Council of Queen Victoria's Jubilee Institute for Nurses.

Twenty-first Annual Report (for 1913) of the London Spectacle Mission Society.

RATES OF SUBSCRIPTION: Twelve months, 6/6; Six months, 4/-; Three months, 2/-, post free to any address in the United Kingdom. Abroad, Twelve months, 8/8; Six months, 5/-; Three months, 2/6, post free.—Address The Subscription Dept., "THE HOSPITAL," The Hospital Building, 28/29 Southampton Street Strand, London, W.C.

